# Artificial intelligence model comparison for risk factor analysis of patent ductus arteriosus in nationwide very low birth weight infants cohort

**DOI:** 10.1038/s41598-021-01640-5

**Published:** 2021-11-16

**Authors:** Jae Yoon Na, Dongkyun Kim, Amy M. Kwon, Jin Yong Jeon, Hyuck Kim, Chang-Ryul Kim, Hyun Ju Lee, Joohyun Lee, Hyun-Kyung Park

**Affiliations:** 1 Department of Pediatrics, Hanyang University College of Medicine, 222 Wangsimni-ro, Seongdong-gu, Seoul, 04763 Korea; 2 Department of Electrical and Electronic Engineering, Hanyang University, 55 Hanyangdaehak-ro, Sangnok-gu, Ansan, 15588 Korea; 3 Artificial Intelligence Convergence Research Center, Hanyang University ERICA, Ansan, 15588 Korea; 4 Department of Medical and Digital Engineering, Hanyang University, 222 Wangsimni-ro, Seongdong-gu, Seoul, 04763 Korea; 5 Department of Thoracic and Cardiovascular Surgery, Hanyang University, 222 Wangsimni-ro, Seongdong-gu, Seoul, 04763 Korea

**Keywords:** Paediatric research, Congenital heart defects, Machine learning, Risk factors

## Abstract

Despite the many comorbidities and high mortality rate in preterm infants with patent ductus arteriosus (PDA), therapeutic strategies vary depending on the clinical setting, and most studies of the related risk factors are based on small sample populations. We aimed to compare the performance of artificial intelligence (AI) analysis with that of conventional analysis to identify risk factors associated with symptomatic PDA (sPDA) in very low birth weight infants. This nationwide cohort study included 8369 very low birth weight (VLBW) infants. The participants were divided into an sPDA group and an asymptomatic PDA or spontaneously close PDA (nPDA) group. The sPDA group was further divided into treated and untreated subgroups. A total of 47 perinatal risk factors were collected and analyzed. Multiple logistic regression was used as a standard analytic tool, and five AI algorithms were used to identify the factors associated with sPDA. Combining a large database of risk factors from nationwide registries and AI techniques achieved higher accuracy and better performance of the PDA prediction tasks, and the ensemble methods showed the best performances.

## Introduction

The ductus arteriosus usually exists during fetal periods, when circulation in the lungs and body is normally supplied by the mother; in term infants, the ductus arteriosus becomes functionally closed by 72 h of age^1,2^. Approximately 20–50% of neonates with gestational age (GA) < 32 weeks have the ductus arteriosus on day 3 of life^3^, and up to 60% of neonates with GA < 29 weeks have it^2,4^. Patent ductus arteriosus (PDA) in preterm infants results in increased mortality and morbidities, and clinicians should determine whether PDA treatment can increase the chances of survival against the burden of unintended consequences. However, the criteria for symptomatic PDA (sPDA) and methods/timing of PDA treatment remain controversial depending on the clinical setting^5–7^. This controversy stems from the subjectivity in radiologic findings and clinical judgment of many other neonatal diseases overlapping PDA. Additionally, the controversy increases further if an insufficient workforce exists, such as the lack of pediatric cardiologists or skilled neonatologists. Additionally, artificial intelligence (AI) produces consistent and unbiased results without being affected by fatigue or emotions. Previous studies have proposed methodologies to screen for the presence of PDA in infants by analyzing phonocardiograms using AI techniques^8,9^. The findings revealed that AI techniques outperformed human clinicians^9^. Along with the high performance, explaining the predicted results of AI through risk factor analysis is possible^10^. Therefore, AI can provide a more objective diagnosis by analyzing the factors necessary to classify a patient as sPDA.

AI is the ability of a computer to simulate human intelligence based on substantial amounts of data, sophisticated algorithms, and high computational power^11^. AI can be classified into supervised learning, unsupervised learning, semisupervised learning and reinforcement learning, depending on the problem and dataset^12^. Al technologies are growing in use in the fields of imaging, diagnosis, therapy selection, risk prediction, disease stratification, and precision medicine^13^. For example, prior studies have predicted the risks of heart transplantation and in-hospital mortality by supervised learning^14,15^. More recently, an ensemble model aggregating four different classifiers has been developed to predict agitation in invasive mechanical ventilation patients^16^. Along with advances in the accuracy of AI analytics, methodologies for explainable AI (XAI) have also evolved. XAI accounts for the rationale underlying the decision-making process and shows which risk factors contribute the most to the decision-making process^10^. This study is the first report to analyze the perinatal risk factors for preterm sPDA cases registered in a nationwide cohort database and to suggest the feasibility of supervised learning-based AI in newborn screening for this disease. To date, respiratory distress syndrome (RDS), birth weight, sex, and gestational age have been deemed risk factors for sPDA^17,18^. In addition to the factors commonly considered by clinicians, we tried to determine whether various factors affect PDA; thus, the model was configured to include all the registered variables as much as possible. Ultimately, we tried to diagnose sPDA as soon as possible using only perinatal factors without imaging findings.

This study aimed to investigate the perinatal risk factors leading to sPDA and sPDA treatments for very low birth weight (VLBW) infants in a nationwide cohort registry and to compare the performance of AI analysis with that of conventional analysis. This study may support the idea that an integrated combination of Al and conventional analysis can synergistically aid clinical risk prediction and therapy selection in medicine.

## Methods

### Study design

#### Patients and data collection

In this study, we derived data from infants registered in the Korean Neonatal Network (KNN), a nationwide prospective web-based registry of VLBW infants. These data were collected from patients admitted to 74 neonatal intensive care units (NICUs) in Korea and analyzed retrospectively. The KNN registry was approved by the institutional review board of each participating hospital. Informed consent was obtained from the parents of each infant prior to participation in the KNN registry. All the methods were performed in accordance with the relevant guidelines and regulations. This study was supported by the Korea Centers for Disease Control and Prevention (2019-ER7103-01)^19^ and was approved by the Hanyang University Institutional Review Board (IRB No. 2013-06-025-043).

**Figure 1:**
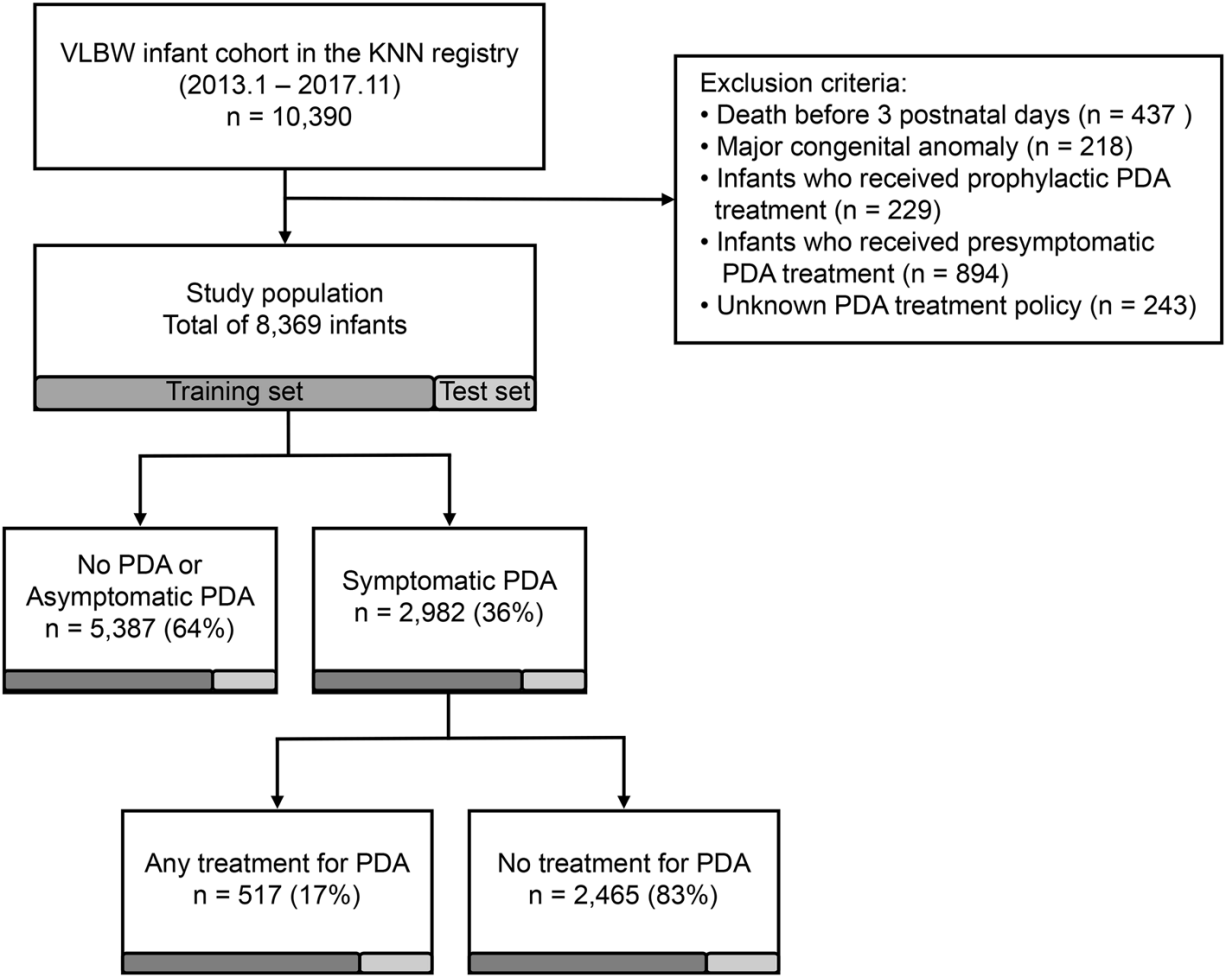
The study population was identified using a subsequent flowchart of the study. VLBW infants, very low birth weight infants; KNN, Korean Neonatal Network; PDA, patent ductus arteriosus.

The cohort data comprised 10,390 VLBW infants born between January 5, 2013, and November 19, 2017, weighing less than 1500 g. Infants who had died before three postnatal days since the confirmation of sPDA was impossible, those who had received prophylactic or presymptomatic PDA treatment, and those who had major congenital anomalies were excluded. Infants with missing or unknown PDA treatment policies were also excluded. After this exclusion process, 8,369 infants from the KNN were eligible for sPDA prediction and risk factor analysis. After the group without PDA was excluded, the data of 2,982 patients remained and were used to analyze the treatment-determining factors of sPDA (Fig. 1).

#### Definitions

According to the PDA treatment strategy, KNN classified the population as follows: group 1, prophylactic PDA treatment without clinical symptoms or abnormal echocardiographic findings; group 2, PDA treatment performed without clinical symptoms due to PDA although PDA was confirmed by echocardiography; group 3, sPDA treatment as PDA treatment performed because of clinical symptoms due to PDA; group 4, only conservative and supportive treatment although with clinical symptoms due to PDA; group 5, asymptomatic PDA or spontaneously closed PDA (nPDA); group 6, missing or unknown PDA treatment policy. sPDA was defined as the presence of more than 2 of the following 5 clinical symptoms with/without echocardiographic confirmation of a large left-to-right ductal flow: (1) a systolic or continuous murmur; (2) a bounding pulse or hyperactive precordial pulsation; (3) hypotension; (4) respiratory difficulty; and (5) evidence from a chest radiograph (pulmonary congestion, cardiomegaly). According to the therapeutic strategies for PDA registered in the KNN database, we further stratified the sPDA group into the following two subgroups to compare the risk factors in the treatment and nonintervention groups that mediated sPDA closure: treated group (sPDA_tx), comprising patients who had received any treatment for sPDA; untreated group (sPDA_nontx), comprising patients who had received only conservative treatment or no treatment for sPDA. The term “treatment” refers to medication (indomethacin, ibuprofen, and other NSAIDs) or ligation.

### Artificial intelligence analysis

#### Dataset and preparation

The obtained cohort data included the following (1) 23 factors related to the prenatal environment and pregnancy; (2) 21 factors associated with delivery and the period immediately after birth; and (3) 3 factors recorded after birth in the clinical data. In the sPDA_tx group, sepsis and fungal infection included only cases diagnosed earlier than the date of treatment initiation. Except for factors with an ambiguous causal relationship with PDA, all the data from the KNN were used to the greatest extent possible. Each factor was classified as a continuous, ordinal, or nominal variable. The details of each risk factor and abbreviations used in this study are presented in Supplementary Table 1. Some features contained missing values. However, instead of removing missing data, we replaced all occurrences of missing values to include as many variables as possible in the analysis (Supplementary Table 2). The imputation was conducted using the medians of numerical variables and modes of nominal variables. To prevent unseen information from being used in the training process, the median or mode was calculated using only the training set and then imputed to the same values as the test data.

#### Artificial intelligence algorithms

Five classical AI algorithms (AAs) were selected in this study because their specific properties are appropriate for risk prediction analyses: a random forest (RF), a decision tree-based theory used to avoid overfitting; a light gradient boosting machine (L-GBM), a low-bias model formed by combining sequential weak models with a light computational algorithm; a multilayer perceptron (MLP), a feedforward artificial neural network that has excellent pattern extraction capability; a support vector machine (SVM), a model optimized exploiting the kernel trick for highly complex problems in cases where linear separation is not possible; and k-nearest neighbors (k-NN), which perform classification based on most of the nearby data points. Among these AAs, the RF and L-GBM are decision tree-based ensemble models. The AAs used in the present study have been previously used to predict hypertension and cardiovascular risk^12,20^. Further detailed information concerning these AAs is provided in Supplementary Methods 1 and 2.

#### Hyperparameter optimization

The study population was divided into a training set and a test set at a ratio of 80:20 using stratified random sampling^21^. To avoid AA methods overfitting the test set, we applied fivefold cross-validation to the training set in validating procedure, and the area under the receiver operating curve (AUC) was averaged over all the data fold sets^22,23^. The stratified cross-validation method ensures that each training and test fold has a similar distribution of outcomes with the entire dataset to reduce bias in the training and evaluating processes. Additionally, cross-validation improves generalization performance by estimating the performance of the model by averaging the results of multiple validations^24^. We used a grid search to find the optimal hyperparameter that maximizes the AUC by performing cross-validation on all possible hyperparameter combinations.

A significant difference was found in the number of positive and negative classes in the study population. If the data are unbalanced, the problem arises that the model gives a higher weight to the majority class; thus, the sensitivity to the minority class would be reduced. We resolved the class imbalance using the synthetic minority oversampling technique (SMOTE), an oversampling method in which a new minority class sample is synthesized by adding a random value to the sample of the original minority class^25^. We evaluated the performance of the AAs using the AUC and accuracy metrics, and 95% confidence intervals (CIs) were calculated using bootstrapping^26^. We implemented the AAs using Python 3.8.5 (Python Software Foundation, https://www.python.org/) and a compatible package—i.e., Scikit-Learn version 0.24 (https://scikit-learn.org/)^27^.

#### Shapely additive explanation

After training the AAs, we analyzed the associations between the risk factors and outcomes. AI classifiers are black boxes that do not reveal their internal working processes, making it challenging to understand the associations between specific factors and decisions^28^. To give clinicians a convincing reason to trust the decisions, we used a game theory-based AI interpretation method called SHAP (Shapely Additive explanations)^29^. SHAP is a leading algorithm to identify the main risk factors that drive the decisions of a model. The SHAP value of each factor was calculated as the average difference in the prediction probabilities between the combinations of risk factors in which the target risk factor was included and not included. Because of their computational nature, SHAP values ​​can be positive or negative depending on the side to which the given risk factor pushes the model’s predictions.

### MLR analysis

We also evaluated the predictive accuracies of the examined risk factors using a conventional analysis. A multiple logistic regression (MLR) approach was used as the reference method, and the raw data were stratified into a binomial distribution. Variables with a threshold p-value of 0.15 were selected to remain in the model according to backward selection, starting with the full model, and all the regression coefficients were tested using Wald statistics at α = 0.05. The effects whose p-values were less than 0.05 were regarded as significant. The goodness of fitness of the final model was tested using Hosmer–Lemeshow’s method at α = 0.05. The identification of significant effects was based on SAS 9.4 (SAS, Inc., NC, USA, https://www.sas.com/), and prediction analyses were performed using Python 3.7.5 (https://www.python.org/).

### Interpretation of the correlations among the factors

In addition to the main AI-based risk factor analysis, we analyzed the statistical correlations among all the risk factors and outcomes. A correlation matrix was calculated using formulas such as Spearman’s rank, point biserial coefficients, and pi coefficients, depending on the type of variable (continuous, ordinal, nominal), across the entire study cohort (n = 8369). The correlation results were visualized as dendrograms, heatmaps and networks through a hierarchical clustering process using the Ward distance method and the Force Atlas function of the gephi 0.9.2 program (https://gephi.org/)^30–32^.

## Results

### Study population

A total of 10,390 infants born between January 5, 2013, and November 19, 2017 met the KNN’s inclusion/exclusion criteria for the original cohort. Among them, 2,982 (35.6%) patients had sPDA, and 5,387 (64.4%) patients did not. Among those with sPDA, 2465 (82.7%) were treated, and 517 (17.3%) were not treated (Table 1). The same variables were collected for these two study populations.

**Table 1:** Demographic Characteristics of the Study Population (N = 8369).

Characteristic	N (%)	Mean ± SD
**Gestational age (weeks)**		29.1 ± 2.9
< 26	1258 (15.0)	
26–29	2870 (34.3)	
30–33	2381 (28.5)	
34–37	530 (6.3)	
≥ 37	1330 (15.9)	
**Birth weight (g)**		1105.1 ± 276.6
< 500	131 (1.6)	
500–999 g	2736 (32.7)	
1000–1500 g	5502 (65.7)	
Birth height (cm)		36.7 ± 3.6
Birth head circumference (cm)		26.1 ± 2.4
Male sex	4232 (50.6)	
Multiple births (≥ 2)	2935 (35.1)	
Cesarean section	1798 (21.5)	
Grouping by PDA status		
**Symptomatic PDA (sPDA)**	2982 (35.6)	
With any treatment^a^ (sPDA_tx)	2465 (82.7)	
Without treatment (sPDA_nontx)	517 (17.3)	
Asymptomatic PDA or spontaneously closed PDA (nPDA)	5387 (64.4)	

SD, standard deviation; PDA, patent ductus arteriosus.

^a^ Treatments for PDA included medications, such as indomethacin and ibuprofen, as well as ligation surgery.

### Prediction Performance (AI versus MLR)

We compared the prediction performance of the MLR, RF, L-GBM, MLP, SVM and k-NN. The performances of predicting sPDA/nPDA and sPDA_tx/sPDA_nontx were separately measured. The sensitivity, specificity, accuracy and AUC of the MLR and each AA are presented in Table 2. L-GBM achieved the highest performance at predicting sPDA/nPDA in terms of accuracy (0.77 [95% CI, 0.75–0.79]), AUC (0.82 [95% CI, 0.80–0.84]) and specificity (0.84 [95% CI, 0.81–0.86]), and MLR performed best with sensitivity (0.85 [95% CI, 0.83–0.87]). The RF model achieved the best accuracy (0.85 [95% CI, 0.82–0.88]), AUC (0.82 [95% CI, 0.77–0.86]) and sensitivity (0.97 [95% CI, 0.96–0.99]) in determining sPDA_tx and the next best results achieved by the L-GBM and MLR models. The worst model in predicting sPDA and sPDA_tx was the k-NN with all the metrics. The receiver operating characteristic curves are shown in Supplementary Fig. 1.

**Table 2:** Performance Metrics of the Algorithms for Predicting sPDA and sPDA_tx, mean values (95% CI).

	sPDA				sPDA_tx			
	Accuracy	AUC	Sensitivity	Specificity	Accuracy	AUC	Sensitivity	Specificity
**MLR**	0.76 (0.74–0.78)	0.81 (0.79–0.83)	0.85 (0.83–0.87)	0.60 (0.58–0.62)	0.85 (0.82–0.87)	0.78 (0.74–0.81)	0.85 (0.28–0.32)	0.98 (0.97–0.99)
**RF**	0.76 (0.74–0.78)	0.81 (0.79–0.84)	0.64 (0.60–0.68)	0.83 (0.81–0.85)	**0.85 (0.82–0.88)**	**0.82 (0.77–0.86)**	0.97 (0.96–0.99)	0.36 (0.28–0.45)
**L-GBM**	**0.77 (0.75–0.79)**	**0.82 (0.80–0.84)**	0.65 (0.61–0.69)	0.84 (0.81–0.86)	0.85 (0.82–0.87)	0.80 (0.76–0.85)	0.93 (0.90–0.95)	0.34 (0.26–0.41)
**MLP**	0.75 (0.73–0.77)	0.81 (0.79–0.83)	0.75 (0.72–0.78)	0.74 (0.72–0.77)	0.77 (0.73–0.80)	0.72 (0.66–0.77)	0.83 (0.80–0.86)	0.52 (0.44–0.61)
**SVM**	0.75 (0.73–0.78)	0.81 (0.79–0.84)	0.76 (0.73–0.79)	0.75 (0.73–0.78)	0.77 (0.74–0.81)	0.77 (0.72–0.82)	0.82 (0.79–0.86)	0.57 (0.48–0.66)
**k-NN**	0.66 (0.64–0.69)	0.74 (0.72–0.77)	0.73 (0.70–0.76)	0.63 (0.60–0.66)	0.67 (0.63–0.71)	0.67 (0.61–0.72)	0.71 (0.67–0.75)	0.49 (0.40–0.58)

sPDA, symptomatic patent ductus arteriosus; sPDA_tx, symptomatic patent ductus arteriosus with any treatment; CI, confidence interval; AUC, area under the receiver operating characteristic curve; MLR, multilinear regression; RF, random forest; L-GBM, light gradient boosting machine; MLP, multilayer perceptron; SVM, support vector machine; k-NN, k-nearest neighbors.

The underlined values denote the highest accuracy and AUC results.

### Variable rankings

**Figure 2:**
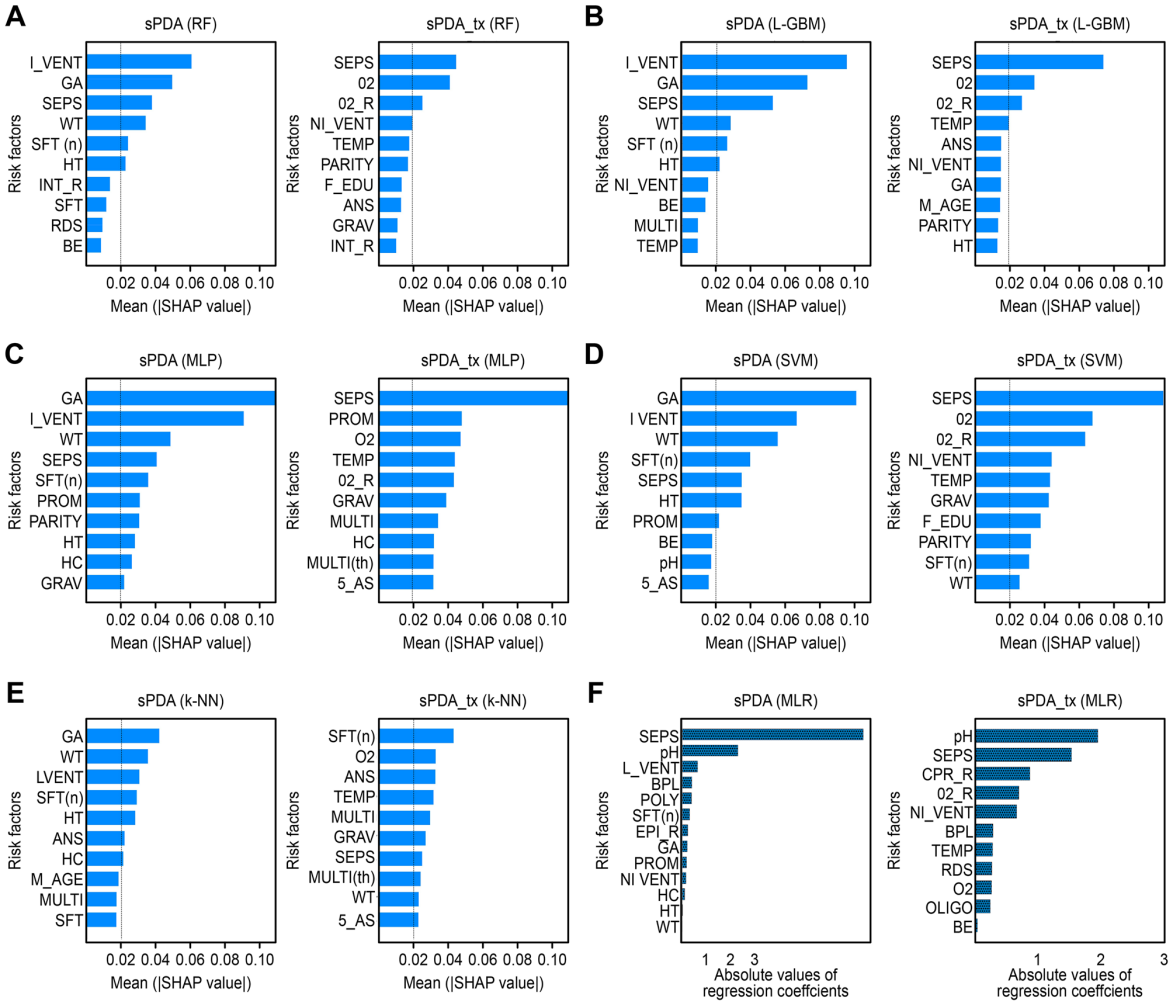
Top 10 factor contributions for sPDA and sPDA_tx prediction derived from each AA and MLR. (**a**) Risk factors for sPDA and sPDA_tx prediction according to the RF. (**b**) Risk factors for sPDA and sPDA_tx prediction according to the L-GBM. (**c**) Risk factors for sPDA and PDA_tx prediction according to the MLP. (**d**) Risk factors for sPDA and sPDA_tx prediction according to the SVM. (**e**) Risk factors for sPDA and sPDA_tx prediction according to k-NN. The risk factors are listed in order of the average absolute SHAP values yielded by each algorithm in the artificial intelligence analysis and were selected based on a *p*-value of 0.05 during the testing procedure; the selected factors are sorted in descending order according to the absolute values of the corresponding regression coefficients in the MLR. Abbreviations: sPDA, symptomatic patent ductus arteriosus; nPDA, asymptomatic PDA or spontaneously closed PDA; sPDA_tx, symptomatic patent ductus arteriosus with any treatment; sPDA_nontx, symptomatic patent ductus arteriosus without treatment; RF, random forest; L-GBM, light gradient boosting machine; MLP, multilayer perceptron; SVM, support vector machine; k-NN, k-nearest neighbors; MLR, multiple logistic regression. The abbreviations for all the factors are shown in Supplementary Table 1.

The important factors for the AI classifiers were ranked by the average absolute SHAP values​​, and Fig. 2 lists up to 10 important risk factors for each model. We considered factors with SHAP values greater than 0.20 as important factors and presented those risk factors for each model in Table 3. These procedures were performed separately for sPDA and sPDA_tx. The full rankings of the variables in the AI analysis are shown in Supplementary Table 2.

**Table 3:** Top significant variables for sPDA and sPDA_tx Prediction.

	Standard	Artificial intelligence algorithms				
	MLR^a^	RF	L-GBM	MLP	SVM	k-NN
sPDA vs. nPDA	*SEPS*	*I_VENT*	*I_VENT*	GA	GA	GA
	pH	GA	GA	*I_VENT*	*I_VENT*	WT
	*I_VENT*	SEPS	SEPS	WT	WT	*I_VENT*
	*BPL*	WT	WT	SEPS	*SFT (n)*	*SFT (n)*
	*POLY*	*SFT (n)*	*SFT (n)*	*SFT (n)*	SEPS	HT
	*SFT (n)*	HT	HT	PROM	HT	ANS
	EPI_R			PARITY	PROM	HC
	GA			HT		
	PROM			*HC*		
	*NI_VENT*			*GRAV*		
sPDA_tx vs. sPDA_nontx	pH	SEPS	SEPS	SEPS	SEPS	*O2*
	SEPS	*O2*	*O2*	*PROM*	*O2*	SFT (n)
	CPR_R	*O2_R*	*O2_R*	*O2*	*O2_R*	ANS
	*O2_R*	*NI_VENT*	*TEMP*	*TEMP*	*NI_VENT*	*TEMP*
	*NI_VENT*			*O2_R*	*TEMP*	MULTI
	BPL			GRAV	GRAV	GRAV
	*TEMP*			MULTI	F_EDU	SEPS
	*RDS*			HC	*PARITY*	MULTI (th)
	*O2*			MULTI (th)	SFT (n)	*WT*
	OLIGO			5_AS	*WT*	5_AS

sPDA, symptomatic patent ductus arteriosus; nPDA, asymptomatic PDA or spontaneously closed PDA; sPDA_tx, symptomatic patent ductus arteriosus with any treatment; sPDA_nontx, symptomatic patent ductus arteriosus without treatment; RF, random forest; L-GBM, light gradient boosting machine; MLP, multilayer perceptron; SVM, support vector machine; k-NN, k-nearest neighbors. The abbreviations for all factors are shown in Supplementary Table 1.

Feature importance describes how relevant a factor is to the model's predictions. In MLR, the feature importance values were selected according to a p-value of 0.05 during the testing procedure. These are listed in descending order as the absolute values of the coefficients for the MLR and as the average absolute SHAP values ​​for the AAs. The variables in italics indicate positive associations between the selected factors and sPDA or sPDA_tx.

^a^ The factor analysis with MLR as the standard reference method.

### Positive/negative correlation analysis

The summary plot of SHAP in Supplementary Fig. 2 shows the quantitative contributions of the top 10 factors for sPDA/nPDA and sPDA_tx/sPDA_nontx) in the AI analysis. For example, we found that invasive mechanical ventilator treatment and the number of administered surfactants were positively associated with sPDA, and gestational age, sepsis, birth weight and birth height were negatively correlated. For sPDA_tx, supplemental oxygen, the need for oxygen supplementation at birth, and noninvasive mechanical ventilator treatment were positively associated with sepsis. Antenatal steroid use was negatively correlated.

### Relationships among risk factors

**Figure 3:**
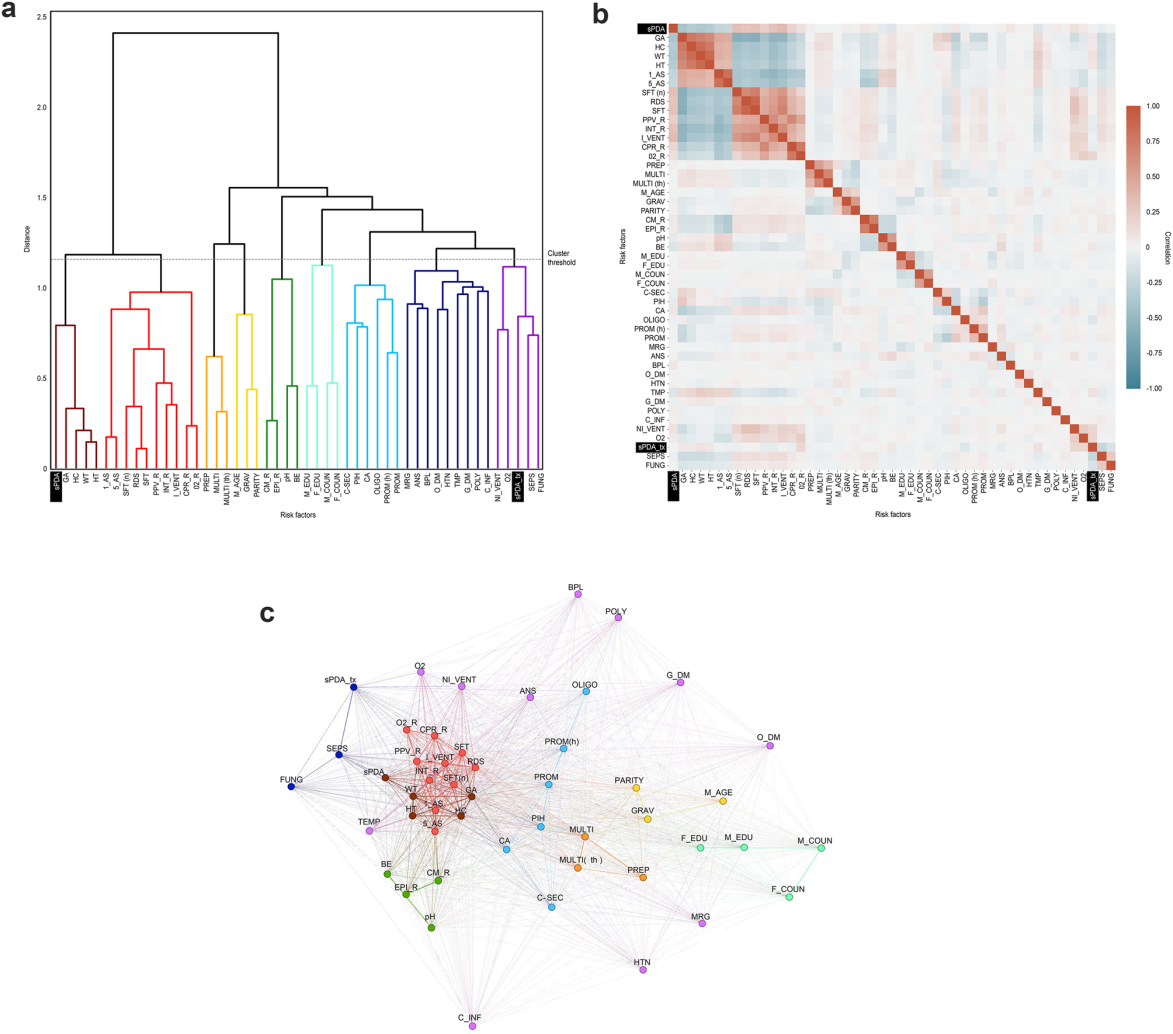
Relationships among the risk factors. (**a**) Dendrogram visualizing hierarchical clustering based on the obtained correlation coefficients. The dendrogram's x-axis comprises sPDA, sPDA_tx and all risk factors, and highly correlated factors are forced to be adjacent through hierarchical clustering. Each horizontal line indicates that the two associated subclusters are merged into one cluster, and the y-height indicates the distance between the two subclusters. We divided the factors into 9 clusters with a threshold of 1.15 and marked each cluster by color. (**b**) Heatmap of the correlation matrix. The x-axis and y-axis of the heatmap follow the arrangement of factors generated by hierarchical clustering, and the correlation coefficients are depicted in red or blue at the intersection of the factors. According to the color bar on the right, red represents a positive correlation, and blue represents a negative correlation. A darker color indicates a higher correlation, while a lighter color indicates a lower correlation. (**c**) Schematic diagram of the relationships among the factors. The circles (nodes) represent the risk factors, connected by the absolute value of the correlation coefficients (edges). In this network, the edges act as attraction forces, bringing highly correlated nodes closer together and pushing less-correlated nodes away from each other. The color of each cluster is the same as that in the dendrogram in (**a**). Abbreviations: sPDA, symptomatic patent ductus arteriosus; sPDA_tx, symptomatic patent ductus arteriosus with any treatment. The abbreviations for all the factors are shown in Supplementary Table 1.

Hierarchical clustering was used to cluster highly correlated factors in a dendrogram (Fig. 3a). sPDA was clustered with the gestational period, birth height, birth weight, and birth head circumference. sPDA_tx was clustered with sepsis and fungal infections, and its cluster was closest to the cluster comprising noninvasive mechanical ventilation, oxygen inhalation, and birth temperature, among others.

According to the heatmap (Fig. 3b), sPDA was negatively correlated with the gestational period, birth height, birth weight, Apgar score, and head circumference. By contrast, sPDA was positively correlated with surfactant administration, positive airway pressure, and endotracheal intubation. sPDA_tx showed a negative correlation with sepsis and fungal infection and a positive correlation with noninvasive mechanical ventilation and oxygen inhalation.

sPDA was relatively close to the Apgar score, physical measurement, resuscitation, and ventilator treatment factors (Fig. 3c). Thus, whether those factors were positively or negatively correlated, they were highly correlated. However, parental factors were far from sPDA, indicating that they were not correlated with sPDA. sPDA_tx was located near sepsis and oxygen supplementation. The factors that were highly correlated with sPDA or sPDA_tx are also shown as important factors in Table 3. Therefore, the important factors derived from the AAs were somewhat consistent.

## Discussion

The analysis of risk factors for symptomatic PDA and determination of PDA treatment in VLBW infants using AI showed higher accuracy and better performance than the conventional analysis. The ensemble model showed a better prediction accuracy and AUC than the other methods when the performances of the models were evaluated. Ultimately, conventional analysis and AI analysis were incorporated to create a new diagram containing the relationships between each factor and sPDA to allow medical staff to intuitively apply these results in actual clinical practice.

According to a relatively recent large-scale study conducted in another country, RDS, birth weight, female sex, gestational age, and the 5-min Apgar score were suggested as risk factors for sPDA in preterm infants^17^. In a study that analyzed 18 factors for hemodynamically significant PDA in preterm infants within 22–29 weeks of gestational age, a lower gestational age, pregnancy-induced hypertension (PIH), and surfactant use were analyzed as risk factors^18^. In the present study, a very low gestational age, a low birth weight and height, and the number of administered surfactants showed close correlations with sPDA. However, the presence of RDS and PIH did not significantly affect the prediction of sPDA. Instead, in the case of invasive ventilator care, a clear prediction of sPDA was shown. In the case of sepsis, the opposite result was obtained with MLR and other AAs. Regarding the national data used in this study, the definition of sepsis was limited to patients who had positive blood cultures or had received more than 5 days of systemic antibiotic treatment. Therefore, the definition of neonatal sepsis is unclear^33^, and the inability to include culture-negative sepsis in particular led to the creation of statistical bias.

For the prediction of sPDA treatment, AI showed very high accuracy and good performance. Because no study has determined the presence or absence of treatment for sPDA, knowing exactly which model selects the most accurate factors is impossible. Generally, a relatively high probability exists that PDA will be treated with absent sepsis when supplemental oxygen is provided during hospitalization (O2) or is needed at birth (O2_R) and when noninvasive ventilator care is required (NI_VENT). When examining each analysis method in detail, some differences were found. These differences were considered due to differences in the strategies used at various hospitals, even in cases in which the sPDA situations were the same and when the time point of sPDA and that at which treatment was started were different.

In recent years, AI has been applied and used in various fields beyond simple engineering domains, and advances in machine learning have begun to affect real-world decisions in many areas, including politics, economics, finance, and medicine^34,35^. The applications of AI in health care include image analysis, treatment, the diagnosis and prognosis of diseases, health care, the improvement of medical administration and management systems, and drug development. In some studies, AI has shown sufficient or rather high disease risk prediction ability compared with existing models^20,36^.

To our best knowledge, this study is the first to use AAs to predict sPDA and sPDA_tx and to analyze the main risk factors for sPDA using large-scale cohort data comprising only electronic records and structured factors (excluding images). The proposed AI classifiers can classify patients well, even when nonlinear relationships exist in the data. This nonlinear characteristic makes it difficult to interpret the prediction processes of AI classifiers. However, by introducing a game-theoretical contribution-based explanation algorithm (SHAP), we identified the main factors. That the AI models’ performances exceeded AUCs of 0.8 and that the main factors were identified using a mathematically fair explanation method support the study's validity. Additionally, the main factors derived from AI and those that were statistically highly correlated with the outcomes coincided, enhancing the consistency of the AI and correlation analysis results.

According to the SHAP interpretation, too many factors were considered in the case of analysis other than the ensemble models, indicating that these models overfitted trivial factors and had lower performances (Table 3). However, the tree-based ensemble models achieved the highest performances because they are designed to overcome overfitting. Ensemble learning has been demonstrated as a solution to construct balanced datasets to enhance prediction performance.

However, AI analysis still has some limitations, such as representation, accuracy, and homogeneity, which occur during the data collection process^37^, and the nature of self-extracting data from large datasets makes it difficult to determine how an AI method produces results and why errors occur^38,39^. Overreliance on AI models when making decisions or analyzing images may lead to automation bias, and it is difficult to analyze the basis of a given judgment^40^. Furthermore, because an SHAP value is a measure of the corresponding factor's contribution to the model result, predict the amount of change induced in a model’s prediction based on a change in factor value is impossible. Additionally, the data collected by the KNN are not focused on PDA; thus, limited factors are included. The lack of information, including vital signs, may reduce the performance of AI. In addition to the variables studied in this study, better results will be obtained if more individual data, such as chest radiographs and echocardiographs, are collected for future studies. Thus, AI remains indispensable for use by medical staff who treat patients directly in clinical practice.

To overcome the abovementioned limitations, this study attempted to enhance objectivity by conducting integrated analysis of MLR, which has been widely used, and AAs. To consider the effects of multicollinearity, Fig. 2c was used to understand the interrelationships among the factors so that the factors most strongly correlated with PDA could recognize the influence of one another more readily than they recognize their influence on PDA.

Using the present study, a follow-up study is planned that prospectively analyzes risk factors and applies management for PDA. Additionally, although this study used existing AAs, in future studies, we will try to study more advanced techniques to improve generalization performance, analyze risk factors by developing a new model explanation method that detects the amounts of changes in risk factors, analyze the relationships between treatment methods and long-term prognosis according to the timing of a given treatment and propose the best treatment policy.

This AI analysis using a nationwide cohort registry is the first study of VLBW infants in the NICU. We evaluated risk factor variables associated with and potentially causally linked to sPDA and sPDA_tx and showed that the ensemble models (RF and L-GBM) were the best among the examined AAs at predicting specific disease development trends, yielding higher accuracy than that of an established risk prediction approach. The use of these readily available online AAs underlined their applicability as an auxiliary means of risk prediction and therapy selection.

## Supplementary Information

Supplementary Information.

Supplementary Tables.

Supplementary Figures.

## Data Availability

According to the Korean Neonatal Network (KNN) Publication Ethics Policy, all information about patients is confidential. The information contained in the data must be protected as confidential, and only available to individuals who have access for the permitted research activity.
