# Supplementary material for: Artificial intelligence model comparison for risk factor analysis of patent ductus arteriosus in nationwide very low birth weight infants cohort
Source: Sci Rep. 2021 Nov 16;11:22353. doi: 10.1038/s41598-021-01640-5 (PMC8595677; doi:10.1038/s41598-021-01640-5)
Supplement: Supplementary file 1 — Supplementary Information. [file 41598_2021_1640_MOESM1_ESM.docx]

**Supplementary Methods 1. The Five Classical Machine Learning Algorithms Used in this Study**

***Random Forest (RF)***

We used the ensemble.RandomForestClassifier function (https://scikit-learn.org/stable/modules/generated/sklearn.ensemble.RandomForestClassifier.html) in the Scikit-learn package. The RF is an ensemble learning method that uses a bagging algorithm and performs prediction by a majority vote of multiple decision trees that are grown independently during training. Bagging is an abbreviation for bootstrapping aggregation, and each decision tree is grown with an independent dataset created by replacement sampling. Bagging reduces the variance of predictions and avoids overfitting. We set the following hyperparameter grid for the random forest:{n_estimator=[400,700,1000], max_depth= [6,8,10,12], min_sample_split= [5,10,30,50,70,100], min_sample_leaf= [5,10,30,50,70,100]}; the optimal hyperparameters for sPDA prediction were [n_estimator=400, max_depth=12, min_sample_split=5, min_sample_leaf=5]. The parameters set for sPDA_tx prediction were [n_estimator=700, max_depth=10, min_sample_split=5, min_sample_leaf=5]. n_estimator indicates the number of decision trees in an RF classifier. max_depth indicates a constraint on the sizes of individual decision trees. min_sample_split is a constraint on the minimum number of samples required for each tree node. min_sample_leaf is a constraint on the number of terminal leaves in a tree.

***Light GBM (L-GBM)***

We used the lightgbm.LGBMClassifier (<https://lightgbm.readthedocs.io/en/latest/Python-API.html>) function of the lightgbm package’s Python application programming interface (API). L-GBM is a model that improves speed by reducing the number of required computations based on the highly accurate gradient boosting algorithm. Gradient boosting is an algorithm that creates a high-accuracy model by combining decision trees to compensate for errors. During training, the gradient of the error function is used^1^. We limited the max_depth of the trees to 8 to avoid overfitting and mainly adjusted num_leaves. The hyperparameter ranges of lightgbm were {boosting_type= ['gdbt', 'dart'], min_sample_split= [10, 20, 40, 60, 80, 100], num_leaves= [120, 150, 180, 210, 240], subsample= [0.8, 1], learning_rate=[0.05, 0.1]}. The optimal hyperparameter list was [boosting_type=‘dart’, min_sample_split=40, num_leaves=120, subsample=0.8, learning_rate= 0.05] for sPDA prediction and [boosting_type=‘dart’, min_sample_split=100, num_leaves=120, subsample=0.8, learning_rate=0.1] for sPDA_tx prediction. num_leaves indicates a constraint on the maximum number of tree leaves, and the subsample is a ratio of the instances used for training. Many of the hyperparameters are the same as those used for the RF classifier.

***Multilayer Perceptron (MLP)***

We trained a multilayer neural network using neural_network.MLPClassifier (https://scikit-learn.org/stable/modules/generated/sklearn.neural_network.MLPClassifier.html?highlight=mlp#sklearn.neural_network.MLPClassifier). An MLP is a machine learning model that connects perceptrons in multiple layers, imitating biological synapses. It has excellent pattern recognition ability and is capable of solving complex problems. We used stochastic gradient descent and Adam as optimization algorithms for the neural network^2^. The hyperparameter ranges of the MLP were {solver= ['adam', 'sgd], hidden_layer_sizes= [(20, 20), (40, 40), (60, 60), (20, 20, 20), (40, 40, 40), (80, 80, 80), (100, 100, 100), activation= ['logistic', 'tanh', 'relu'], alpha= [0.0001, 0.0003, 0.00006, 0.0009]}. The optimal hyperparameter list for sPDA prediction was [solver = ‘sgd’, hidden_layer_sizes= (60, 60), activation= 'tanh', alpha= 0.0001]; and for sPDA_tx, it was [solver = ‘sgd’, hidden_layer_sizes= (40, 40), activation= 'relu', alpha= 0.0001]. The activation function was a nonlinear operator for determining how often a node sends its signal to the next node. This nonlinear operation makes the representative power of the MLP excellent, enabling complex classification. However, because strong representative power can easily lead to overfitting, it is important to set the regulatory term's coefficient (alpha) appropriately.

***Support Vector Machine (SVM)***

We used the svm.SVC function of the Scikit-learn package (https://scikit-learn.org/stable/modules/generated/sklearn.ensemble.RandomForestClassifier.html). This algorithm assumes that data can be linearly separated by transferring it into a high-dimensional feature space. However, instead of transferring data, this process was implemented as a nonlinear kernel trick (we found that the Gaussian function was the best choice for our data). We controlled the distance from the data to the decision boundary with a parameter gamma. The C factor allowed some data to be present around the boundary of the decision to prevent overfitting. The hyperparameter ranges of the SVM were {C= numpy.linspace(10^-2^, 10^2^, 20), gamma= ['scale', 'auto', 0.1, 1, 10]}. The optimal hyperparameter list for sPDA prediction was [C = 0.483, gamma= 'auto'], and that for sPDA_tx was [C = 1.274, gamma= 'auto'].

***k-Nearest Neighbors (k-NN)***

We used the neighbor.KNeighborsClassifier function of the Scikit-learn package (https://scikit-learn.org/stable/modules/generated/sklearn.neighbors.KNeighborsClassifier.html#sklearn.neighbors.KNeighborsClassifier). This algorithm computes the Euclidean distances between all data points. Then, it selects the k closest points from the target data and observes the outcome classes of the selected data, and the most observed class is presented as the prediction result. The hyperparameter range of k-NN was {n_neighbors= numpy.arange(2, 16)}. The optimal hyperparameter list for sPDA prediction was [n_neighbors = 15], and that for sPDA_tx was [n_neighbors = 15].

**Supplementary Methods 2. Procedures for Machine Learning Classifier Training**

***Stratified Random Sampling and Hyperparameter Optimization***

We divided the study cohort into a training set and a test set by the stratified random sampling method to preserve the proportions of the classes that were observed in the original data. The same thought process was applied for splitting the utilized 5-fold cross-validation sets. The machine learning training process involved several stages. First, we imputed the missing values with the median of the training set. Then, we divided the training set into 5 folds and resolved the imbalance in the data using the synthetic minority oversampling technique (SMOTE) algorithm. Next, we set up a hyperparameter grid and trained each model at every point in the grid. Finally, the optimized hyperparameter with the best cross-validation performance was selected, and the models were retrained on the entire training set. This training process was performed by the GridSearchCV function of the Scikit-learn package.

***SMOTE***

As shown in Figure 1, this study cohort had an imbalanced distribution of sPDA and sPDA_tx instances. If a model is fitted to imbalanced data, it overfits the majority class and exhibits degraded generalization performance for unseen data. Therefore, we generated synthetic data among the minority samples to resolve these problems. We synthesized minority class samples through SMOTE and made the number of minority samples equal to the number of majority samples during the training process^3^.

**Supplementary References**

1. Ke, G. et al. Lightgbm: a highly efficient gradient boosting decision tree. *Adv. Neural Inf. Process. Syst.* **30,** 3146-3154 (2017).

2. Kingma, D. P. & Ba, J. Adam: a method for stochastic optimization. *arXiv:1412.6980* (2014).

3. Chawla, N. V., Bowyer, K. W., Hall, L. O. & Kegelmeyer, W. P. SMOTE: synthetic minority over-sampling technique. *J. Artif. Intell. Res.* **16,** 321-357 (2002).
