# Supplementary material for: Artificial intelligence model comparison for risk factor analysis of patent ductus arteriosus in nationwide very low birth weight infants cohort
Source: Sci Rep. 2021 Nov 16;11:22353. doi: 10.1038/s41598-021-01640-5 (PMC8595677; doi:10.1038/s41598-021-01640-5)
Supplement: Supplementary file 2 — Supplementary Tables. [file 41598_2021_1640_MOESM2_ESM.docx]

**Supplementary Table 1**. Risk Factor Variables and Abbreviations Used in the Analysis

|  | Factors related to the prenatal environment and pregnancy (23 factor variables) | Factors associated with delivery and the period immediately after birth (21 factor variables) | Factors recorded after birth (3 factor variables) |
| --- | --- | --- | --- |
| Continuous variables | Maternal age (M_AGE, years); premature rupture of membranes (PROM(h), hours); gestational age (GA, weeks) | Birth weight (WT, g); birth height (HT, cm); birth head circumference (HC, cm); body temperature at birth (TEMP, ℃); hydrogen ion concentration in the blood within 1 hour after birth (pH); base excess within 1 hour after birth (BE) |  |
| Ordinal variables | Gravida (GRAV); paternal education level (F_EDU); maternal education level (M_EDU); multiple gestation (MULTI); order of multiple births (MULTI(th)); antenatal steroid use (ANS, never administered/incomplete administration/complete administration) | Apgar score at 1 minute (1_AS); Apgar score at 5 minutes (5_AS) | Number of administered surfactants (SFT(n)) |
| Nominal variables | Parity (PARITY); oligohydramnios (OLIGO); polyhydramnios (POLY); maternal country of origin (M_COUN); Paternal country of origin (F-COUN); marital status (MRG, cohabitation/separation); pregnancy process (PREP, natural pregnancy/in vitro fertilization); gestational diabetes mellitus (G_DM); overt diabetes mellitus (O_DM); pregnancy-induced hypertension (PIH); chronic hypertension (HTN); histological chorioamnionitis (CA); premature rupture of membranes (PROM); delivery mode (C-SEC, virginal delivery/cesarean section) | Birth place (BPL, in the hospital/outside the hospital); need for initial resuscitation (CPR_R); need for oxygen supplementation at birth (O2_R); need for cardiac massage at birth (CM_R); epinephrine administration at birth (EPI_R); need for endotracheal intubation at birth (INT_R); need for continuous positive airway pressure at birth (PPV_R); respiratory distress syndrome (RDS); need for surfactant (SFT); invasive mechanical ventilation (I_VENT); noninvasive mechanical ventilation (NI_VENT); supplemental oxygen (O2); congenital infection (C_INF) | Sepsis (SEPS); fungal infection before PDA treatment (FUNG) |

**Supplementary Table 2**. Data and missing values for the variables used in the analysis

| Variables | | No. of patients with valid data entries | No. of missing values | Minimum value in continuous or ordinal variables (No. of patients as negative in nominal variables) | Maximum value in continuous or ordinal variables (No. of patients as negative in nominal variables) |
| --- | --- | --- | --- | --- | --- |
| Continuous variables | M_AGE | 8369 | 0 | 13 | 49 |
|  | PROM(h) | 7996 | 373 | 0 | 8764 |
|  | GA | 8369 | 0 | 21+0 | 36+6 |
|  | WT | 8369 | 0 | 300 | 1499 |
|  | HT | 8026 | 343 | 20 | 49 |
|  | HC | 8007 | 362 | 17 | 38 |
|  | TEMP | 8137 | 232 | 31.3 | 38.7 |
|  | pH | 6398 | 1971 | 6.5 | 7.72 |
|  | BE | 6385 | 1984 | -26 | 15.8 |
| Ordinal variables | GRAV | 8369 | 0 | 1 | 16 |
|  | F_EDU | 4712 | 3657 | 1 | 4 |
|  | M_EDU | 6440 | 1929 | 1 | 4 |
|  | MULTI | 8369 | 0 | 1 | 4 |
|  | MULTI(th) | 8369 | 0 | 1 | 4 |
|  | ANS | 8185 | 184 | 0 | 2 |
|  | 1_AS | 8310 | 59 | 0 | 10 |
|  | 5_AS | 8315 | 54 | 0 | 10 |
|  | SFT(n) | 8369 | 0 | 0 | 9 |
| Nominal variables | PARITY | 8369 | 0 | 5210 | 3159 |
|  | OLIGO | 8369 | 0 | 7246 | 1123 |
|  | POLY | 8369 | 0 | 8272 | 97 |
|  | M_COUN | 8369 | 0 | 8044 | 325 |
|  | F_COUN | 8369 | 0 | 8187 | 182 |
|  | MRG | 8369 | 0 | 8285 | 84 |
|  | PREP | 8369 | 0 | 6419 | 1950 |
|  | G_DM | 8369 | 0 | 7713 | 656 |
|  | O_DM | 8369 | 0 | 8280 | 89 |
|  | PIH | 8369 | 0 | 6667 | 1702 |
|  | HTN | 8369 | 0 | 8181 | 188 |
|  | CA | 6948 | 1421 | 4508 | 2440 |
|  | PROM | 8304 | 65 | 5378 | 2926 |
|  | C-SEC | 8369 | 0 | 1798 | 6571 |
|  | BPL | 8369 | 0 | 8087 | 282 |
|  | CPR_R | 8311 | 58 | 1009 | 7302 |
|  | O2_R | 8311 | 58 | 1597 | 6714 |
|  | CM_R | 8311 | 58 | 7985 | 326 |
|  | EPI_R | 8311 | 58 | 8097 | 214 |
|  | INT_R | 8311 | 58 | 3369 | 4942 |
|  | PPV_R | 8311 | 58 | 1962 | 6349 |
|  | RDS | 8369 | 0 | 2059 | 6310 |
|  | SFT | 8369 | 0 | 2004 | 6365 |
|  | I_VENT | 8369 | 0 | 2313 | 6056 |
|  | NI_VENT | 8369 | 0 | 1913 | 6456 |
|  | O2 | 8369 | 0 | 4894 | 4085 |
|  | C_INF | 8369 | 0 | 8267 | 102 |
|  | SEPS | 8369 | 0 | 7383 | 986 |
|  | FUNG | 8369 | 0 | 8290 | 79 |

The abbreviations of all factors are shown in Supplementary Table 1.

**Supplementary Table 3**. Full Rankings of Important Variables in the Artificial Intelligence Analysis

|  | **RF** | **SHAP value** | **L-GBM** | **SHAP value** | **MLP** | **SHAP value** | **SVM** | **SHAP value** | **K-NN** | **SHAP value** |
| --- | --- | --- | --- | --- | --- | --- | --- | --- | --- | --- |
| **sPDA**  **vs.**  **nPDA** | I_VENT  GA  SEPS  WT  SFT(n)  HT  INT_R  SFT  RDS  BE  5_AS  HC  NI_VENT  pH  TEMP  MULTI  PROM  ANS  OLIGO  PROM(h)  PPV_R  PARITY  1_AS  MULTI(th)  GRAV  O2  M_AGE  CA  M_EDU  PREP  O2_R  PIH  BPL  F_EDU  CPR_R  C-SEC  G_DM  CM_R  EPI_R  M_COUN  FUNG  POLY  F_COUN  HTN  O_DM  C_INF  MRG | 0.061  0.050  0.038  0.034  0.024  0.023  0.014  0.012  0.009  0.009  0.008  0.007  0.007  0.006  0.006  0.006  0.006  0.005  0.004  0.004  0.003  0.003  0.003  0.003  0.003  0.002  0.002  0.002  0.002  0.002  0.002  0.001  0.001  0.001  0.001  0.001  0.001  0.000  0.000  0.000  0.000  0.000  0.000  0.000  0.000  0.000  0.000 | I_VENT  GA  SEPS  WT  SFT(n)  HT  NI_VENT  BE  MULTI  TEMP  PROM  pH  HC  O2  5_AS  ANS  OLIGO  1_AS  M_AGE  PARITY  GRAV  INT_R  PROM(h)  MULTI(th)  RDS  BPL  CA  M_EDU  O2_R  PREP  F_EDU  PIH  G_DM  PPV_R  M_COUN  CM_R  C-SEC  CPR_R  POLY  SFT  FUNG  F_COUN  O_DM  HTN  EPI_R  C_INF  MRG | 0.095  0.073  0.052  0.028  0.026  0.022  0.015  0.013  0.009  0.009  0.009  0.007  0.007  0.007  0.006  0.006  0.006  0.005  0.005  0.005  0.004  0.004  0.004  0.003  0.003  0.003  0.003  0.003  0.002  0.001  0.001  0.001  0.001  0.001  0.001  0.001  0.001  0.000  0.000  0.000  0.000  0.000  0.000  0.000  0.000  0.000  0.000 | GA  I_VENT  WT  SEPS  SFT(n)  PROM  PARITY  HT  HC  GRAV  BE  1_AS  SFT  5_AS  pH  NI_VENT  O_DM  MULTI  TEMP  MULTI(th)  O2  INT_R  ANS  M_AGE  M_EDU  BPL  OLIGO  PPV_R  F_EDU  CA  G_DM  O2_R  PIH  PROM(h)  C-SEC  RDS  CPR_R  F_COUN  PREP  EPI_R  M_COUN  CM_R  POLY  MRG  FUNG  C_INF  HTN | 0.113  0.091  0.049  0.041  0.036  0.031  0.031  0.028  0.027  0.022  0.020  0.020  0.019  0.016  0.016  0.015  0.011  0.011  0.011  0.011  0.010  0.010  0.009  0.009  0.008  0.005  0.005  0.005  0.005  0.004  0.004  0.004  0.003  0.003  0.003  0.002  0.002  0.002  0.001  0.001  0.001  0.001  0.001  0.001  0.000  0.000  0.000 | GA  I_VENT  WT  SFT(n)  SEPS  HT  PROM  BE  pH  5_AS  SFT  PARITY  HC  1_AS  GRAV  INT_R  M_AGE  F_EDU  ANS  MULTI(th)  TEMP  MULTI  NI_VENT  OLIGO  O2_R  O2  CA  PROM(h)  PREP  M_EDU  RDS  PIH  BPL  G_DM  C-SEC  CPR_R  EPI_R  PPV_R  FUNG  F_COUN  HTN  CM_R  POLY  O_DM  MRG  M_COUN  C_INF | 0.101  0.067  0.056  0.040  0.035  0.035  0.022  0.018  0.017  0.016  0.015  0.015  0.013  0.011  0.011  0.008  0.008  0.008  0.007  0.007  0.007  0.006  0.006  0.005  0.004  0.004  0.004  0.004  0.003  0.003  0.003  0.003  0.003  0.002  0.002  0.002  0.001  0.001  0.001  0.000  0.000  0.000  0.000  0.000  0.000  0.000  0.000 | GA  WT  I_VENT  SFT(n)  HT  ANS  HC  M_AGE  MULTI  SFT  5_AS  RDS  pH  1_AS  BE  M_EDU  GRAV  INT_R  F_EDU  O2  PROM  TEMP  MULTI(th)  PARITY  SEPS  C-SEC  CA  NI_VENT  PREP  PIH  PROM(h)  O2_R  PPV_R  OLIGO  CPR_R  M_COUN  O_DM  G_DM  BPL  CM_R  EPI_R  HTN  F_COUN  FUNG  C_INF  MRG  POLY | 0.042  0.035  0.030  0.029  0.028  0.022  0.021  0.018  0.017  0.017  0.016  0.016  0.016  0.014  0.013  0.013  0.013  0.012  0.012  0.011  0.011  0.011  0.010  0.010  0.010  0.009  0.009  0.009  0.006  0.006  0.005  0.005  0.005  0.005  0.003  0.002  0.001  0.001  0.001  0.000  0.000  0.000  0.000  0.000  0.000  0.000  0.000 |
| **sPDA_tx**  **vs.**  **sPDA_nontx** | SEPS  O2  O2_R  NI_VENT  TEMP  PARITY  F_EDU  ANS  GRAV  INT_R  OLIGO  M_EDU  C-SEC  M_AGE  1_AS  WT  SFT(n)  MULTI  GA  5_AS  PPV_R  HT  BE  CA  MULTI(th)  HC  PIH  PREP  F_COUN  pH  PROM(h)  SFT  BPL  CPR_R  RDS  PROM  M_COUN  G_DM  I_VENT  FUNG  CM_R  HTN  EPI_R  POLY  C_INF  MRG  O_DM | 0.045  0.042  0.026  0.020  0.018  0.018  0.014  0.014  0.011  0.011  0.010  0.008  0.006  0.006  0.006  0.006  0.006  0.005  0.005  0.003  0.003  0.003  0.003  0.003  0.002  0.002  0.002  0.002  0.002  0.002  0.001  0.001  0.001  0.001  0.001  0.001  0.001  0.001  0.000  0.000  0.000  0.000  0.000  0.000  0.000  0.000  0.000 | SEPS  O2  O2_R  TEMP  ANS  NI_VENT  GA  M_AGE  PARITY  HT  OLIGO  WT  GRAV  INT_R  PPV_R  5_AS  M_EDU  1_AS  SFT(n)  pH  BE  F_COUN  HC  C-SEC  PROM  MULTI  F_EDU  PROM(h)  CA  BPL  PIH  PREP  MULTI(th)  RDS  G_DM  SFT  M_COUN  CM_R  CPR_R  FUNG  POLY  O_DM  HTN  EPI_R  I_VENT  C_INF  MRG | 0.075  0.035  0.028  0.020  0.016  0.015  0.015  0.015  0.014  0.014  0.013  0.012  0.012  0.012  0.010  0.009  0.009  0.008  0.008  0.007  0.007  0.007  0.006  0.006  0.005  0.005  0.005  0.004  0.004  0.004  0.003  0.002  0.002  0.002  0.001  0.000  0.000  0.000  0.000  0.000  0.000  0.000  0.000  0.000  0.000  0.000  0.000 | SEPS  PROM  O2  TEMP  O2_R  GRAV  MULTI  HC  MULTI(th)  5_AS  WT  HT  BE  SFT(n)  pH  M_AGE  GA  1_AS  PROM(h)  ANS  F_EDU  M_EDU  PPV_R  PIH  PARITY  C-SEC  NI_VENT  CA  INT_R  CM_R  PREP  BPL  OLIGO  EPI_R  F_COUN  CPR_R  RDS  G_DM  I_VENT  SFT  M_COUN  POLY  HTN  C_INF  FUNG  O_DM  MRG | 0.146  0.049  0.048  0.045  0.044  0.040  0.035  0.033  0.032  0.032  0.032  0.032  0.029  0.029  0.028  0.024  0.023  0.021  0.021  0.020  0.019  0.019  0.019  0.017  0.017  0.015  0.014  0.014  0.013  0.011  0.010  0.008  0.007  0.007  0.007  0.005  0.004  0.004  0.003  0.002  0.002  0.001  0.001  0.001  0.001  0.001  0.001 | SEPS  O2  O2_R  NI_VENT  TEMP  GRAV  F_EDU  PARITY  SFT(n)  WT  HC  M_AGE  MULTI  1_AS  5_AS  HT  BE  ANS  PPV_R  MULTI(th)  M_EDU  PROM  GA  OLIGO  pH  CA  INT_R  PROM(h)  BPL  C-SEC  CPR_R  PIH  PREP  F_COUN  I_VENT  CM_R  SFT  EPI_R  HTN  G_DM  M_COUN  POLY  MRG  C_INF  O_DM  RDS  FUNG | 0.130  0.068  0.064  0.044  0.043  0.043  0.038  0.032  0.031  0.026  0.024  0.024  0.020  0.020  0.018  0.017  0.017  0.017  0.016  0.016  0.016  0.016  0.015  0.012  0.011  0.010  0.010  0.009  0.006  0.006  0.005  0.005  0.004  0.003  0.002  0.002  0.002  0.001  0.001  0.001  0.001  0.001  0.001  0.000  0.000  0.000  0.000 | O2  SFT(n)  ANS  TEMP  MULTI  GRAV  SEPS  MULTI(th)  WT  5_AS  BE  1_AS  M_AGE  M_EDU  F_EDU  HT  GA  PROM  PARITY  HC  NI_VENT  O2_R  C-SEC  CA  pH  PREP  PIH  PROM(h)  INT_R  PPV_R  BPL  CM_R  OLIGO  EPI_R  SFT  RDS  I_VENT  G_DM  CPR_R  M_COUN  F_COUN  HTN  POLY  MRG  C_INF  FUNG  O_DM | 0.043  0.033  0.032  0.031  0.029  0.027  0.025  0.024  0.023  0.023  0.022  0.022  0.022  0.021  0.020  0.020  0.019  0.018  0.017  0.016  0.015  0.015  0.015  0.014  0.013  0.011  0.009  0.006  0.006  0.004  0.004  0.004  0.004  0.003  0.002  0.002  0.001  0.001  0.001  0.000  0.000  0.000  0.000  0.000  0.000  0.000  0.000 |

Abbreviations: sPDA, symptomatic patent ductus arteriosus; nPDA, asymptomatic PDA or spontaneously closed PDA; sPDA_tx, symptomatic patent ductus arteriosus with any treatment; sPDA_nontx, symptomatic patent ductus arteriosus without treatment; RF, random forest; L-GBM, light gradient boosting machine; MLP, multilayer perceptron; SVM, support vector machine; K-NN, k-nearest neighbors. The abbreviations of all the factors are shown in Supplementary Table 1.

^a^ Feature importance values describe how relevant a factor is to the model's predictions. These are listed in descending order in terms of the average absolute SHAP values ​​for the artificial intelligence algorithms.
