# Supplementary material for: Artificial intelligence model comparison for risk factor analysis of patent ductus arteriosus in nationwide very low birth weight infants cohort
Source: Sci Rep. 2021 Nov 16;11:22353. doi: 10.1038/s41598-021-01640-5 (PMC8595677; doi:10.1038/s41598-021-01640-5)
Supplement: Supplementary file 3 — Supplementary Figures. [file 41598_2021_1640_MOESM3_ESM.docx]

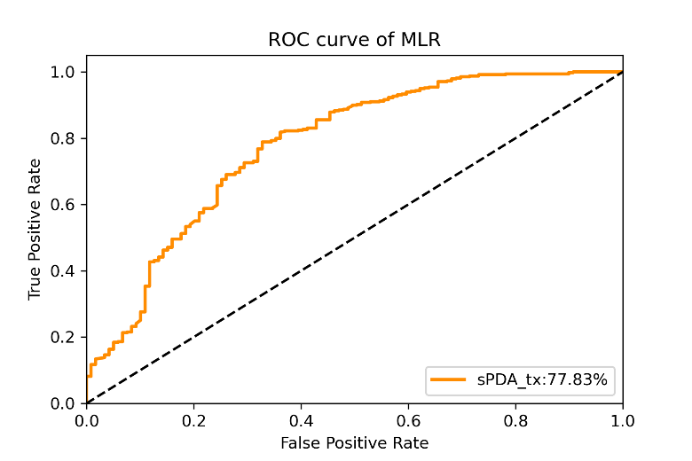
(a) MLR


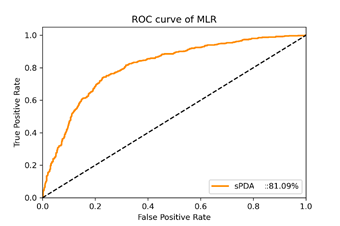


(b) RF


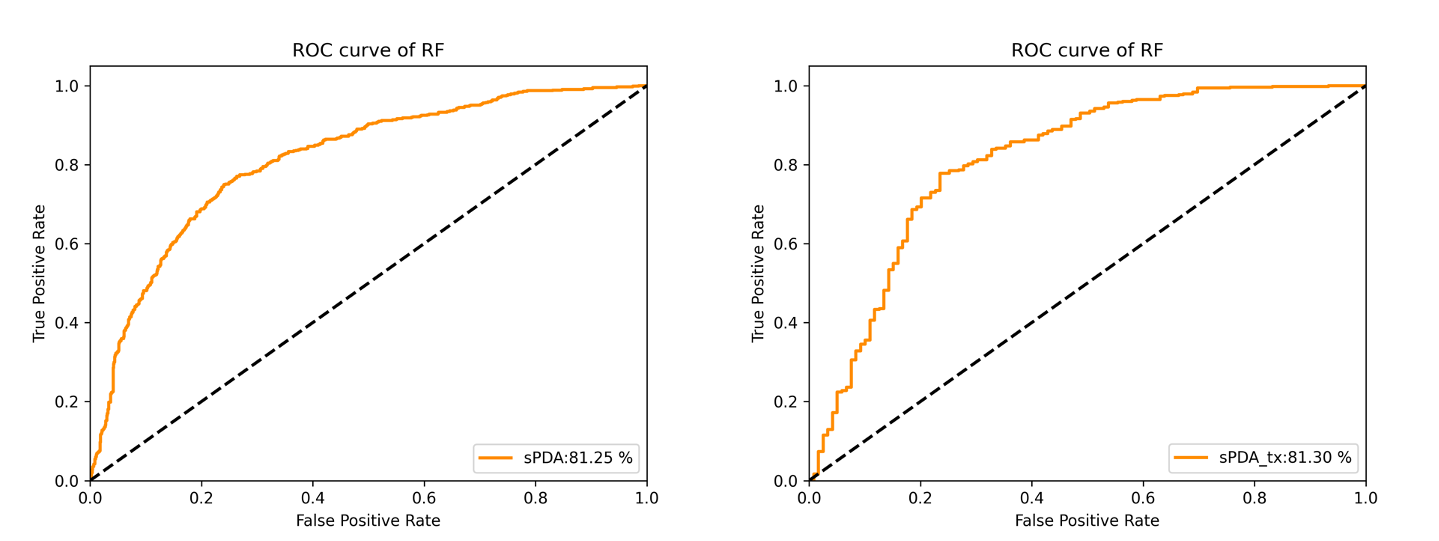


(c) L-GBM


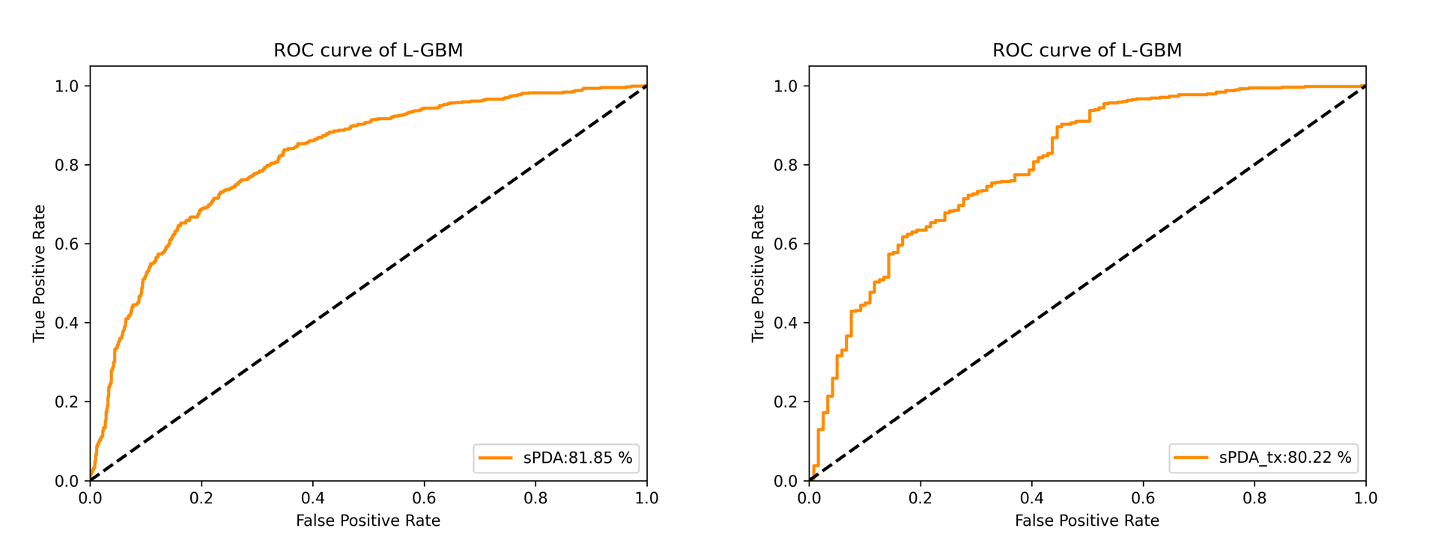


(d) MLP


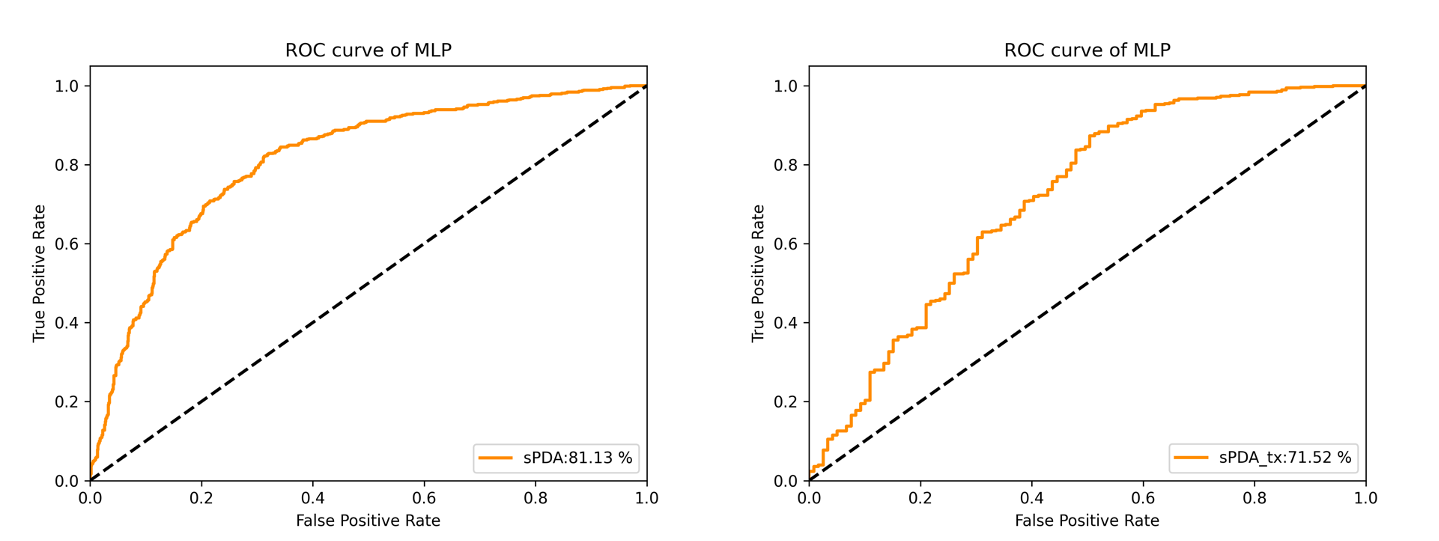


(e) SVM


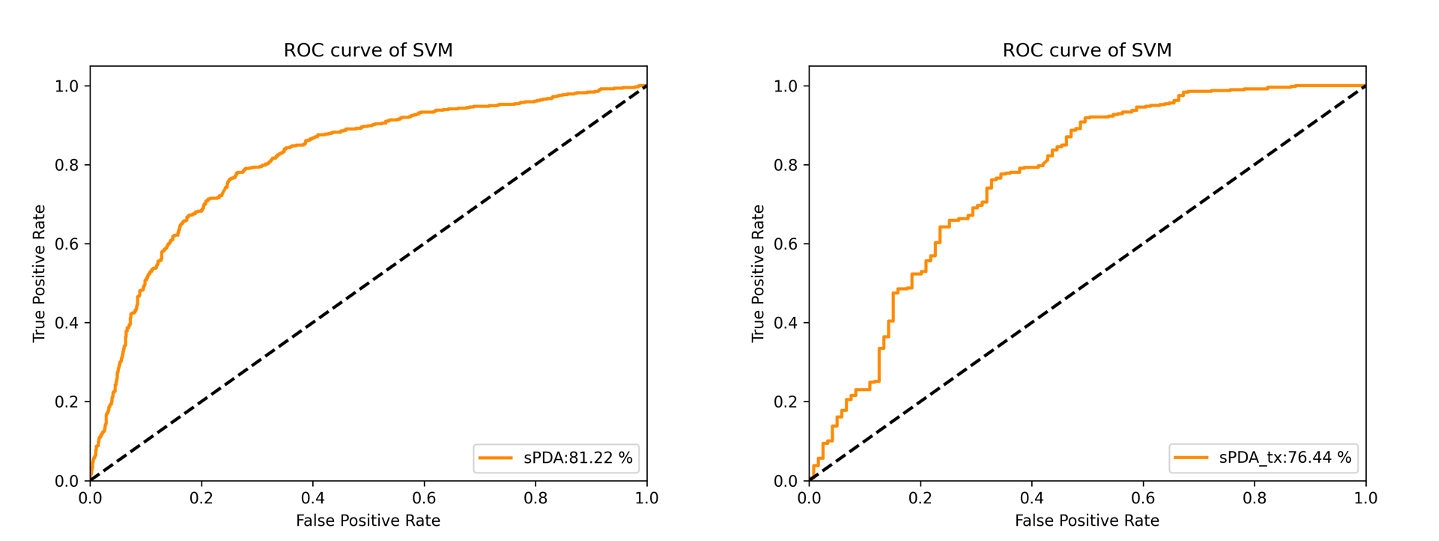


(f) k-NN


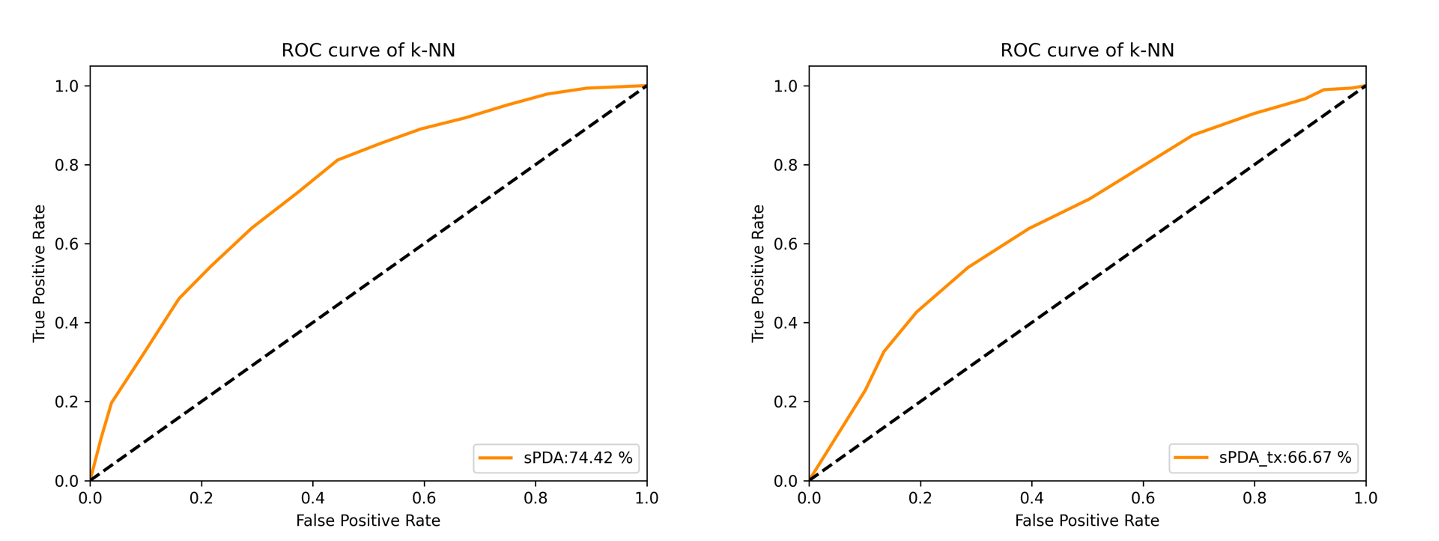


**Supplementary Figure 1**. The area under the receiver operating curve (AUC) for each algorithm with respect to sPDA and sPDA_tx. Abbreviations: ROC, receiver operating curve; PDA, patent ductus arteriosus; sPDA, symptomatic PDA; sPDA_tx, sPDA with any treatment; RF, random forest; L-GBM, light gradient boosting machine; MLP, multilayer perceptron; SVM, support vector machine; k-NN, k-nearest neighbors.

(a) RF


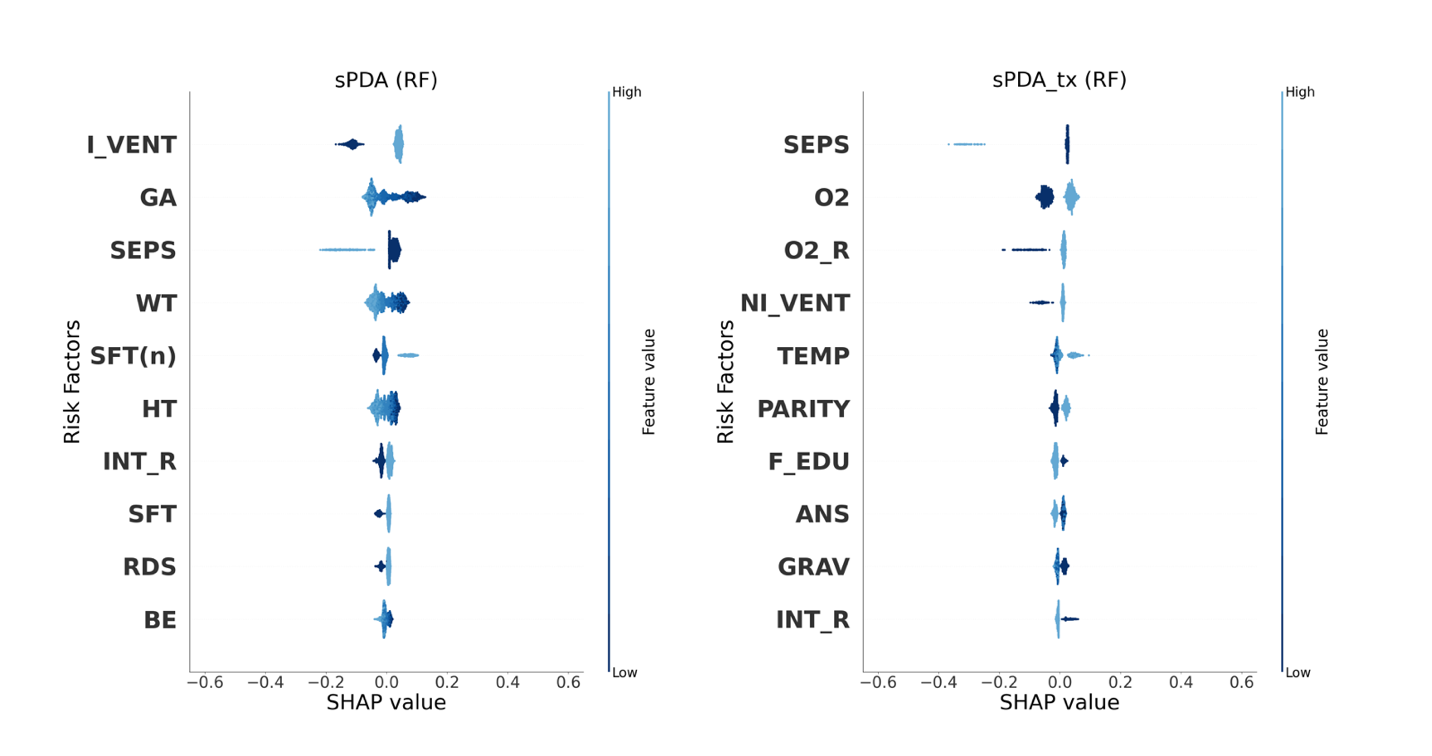


(b) L-GBM


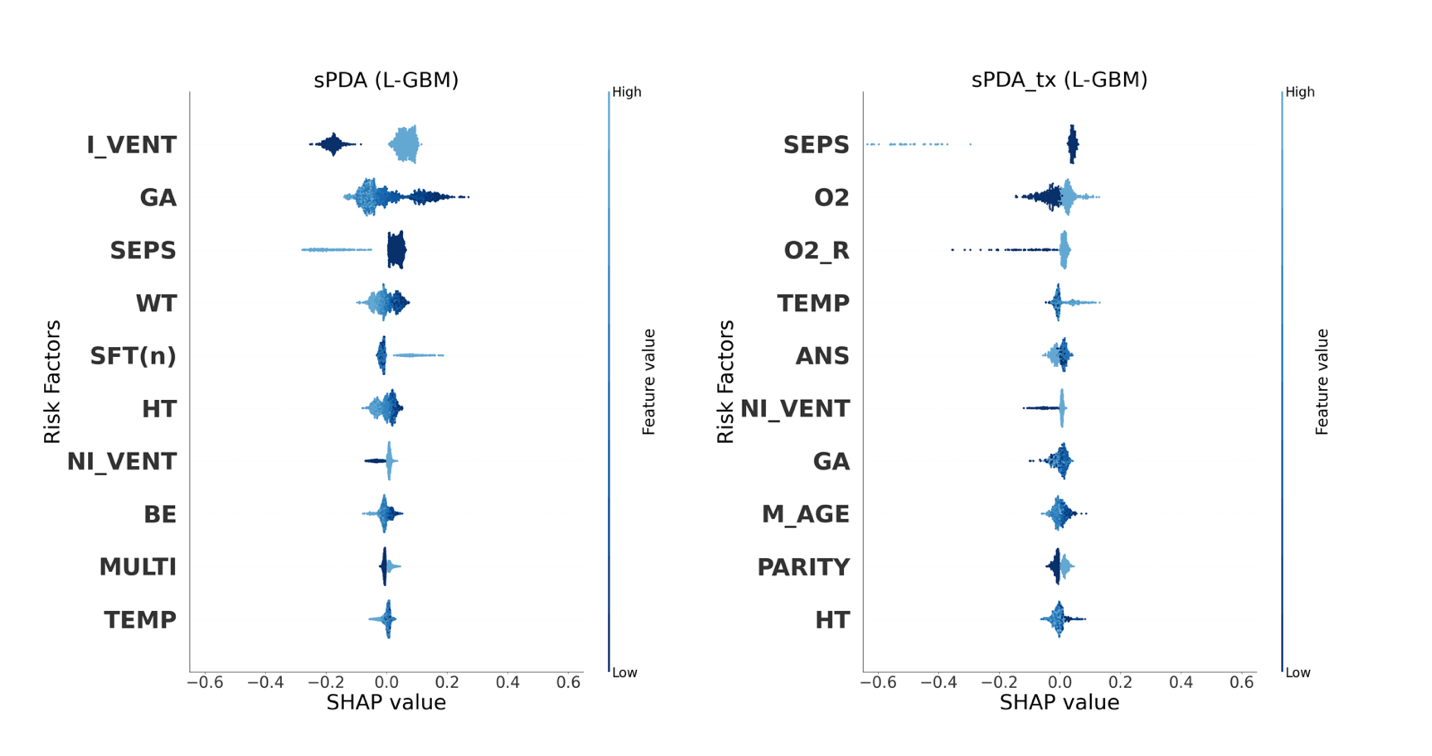


(c) MLP


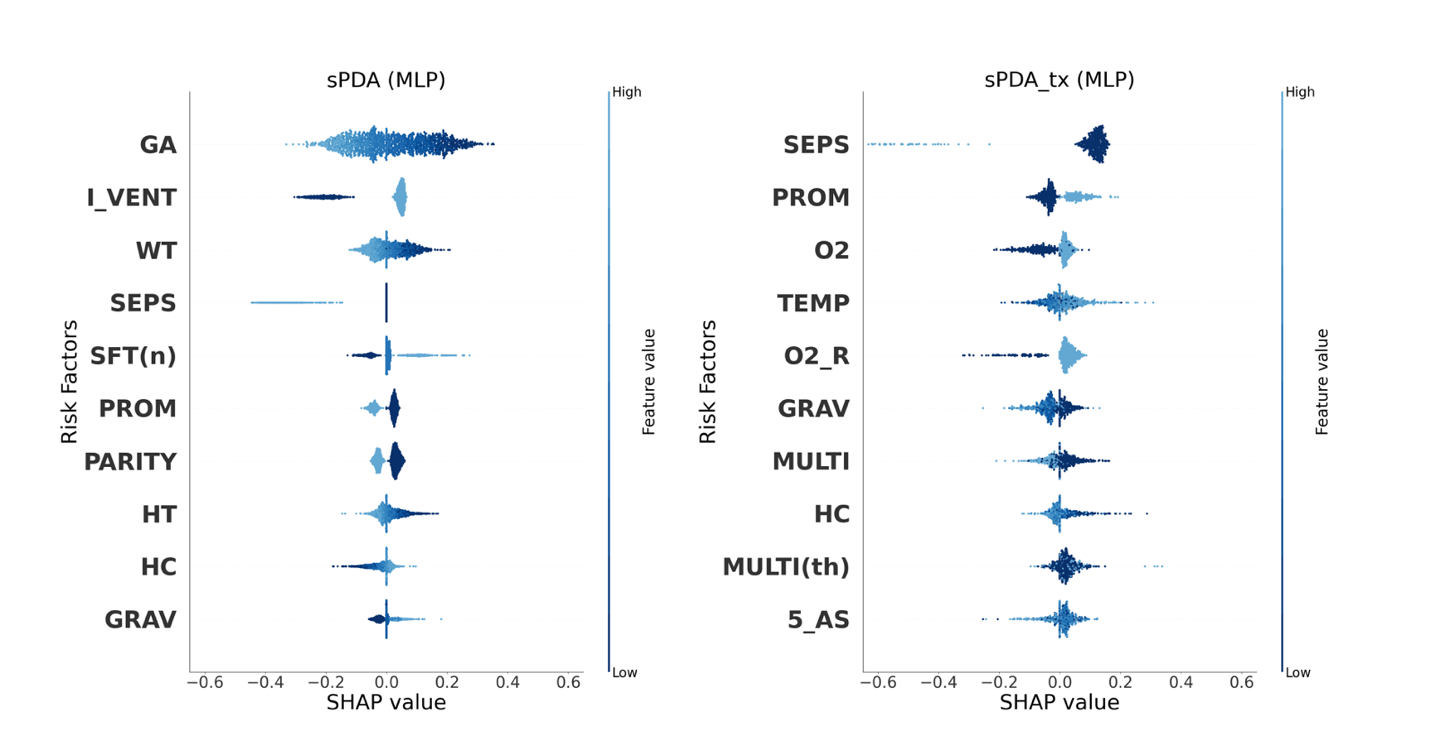


(d) SVM


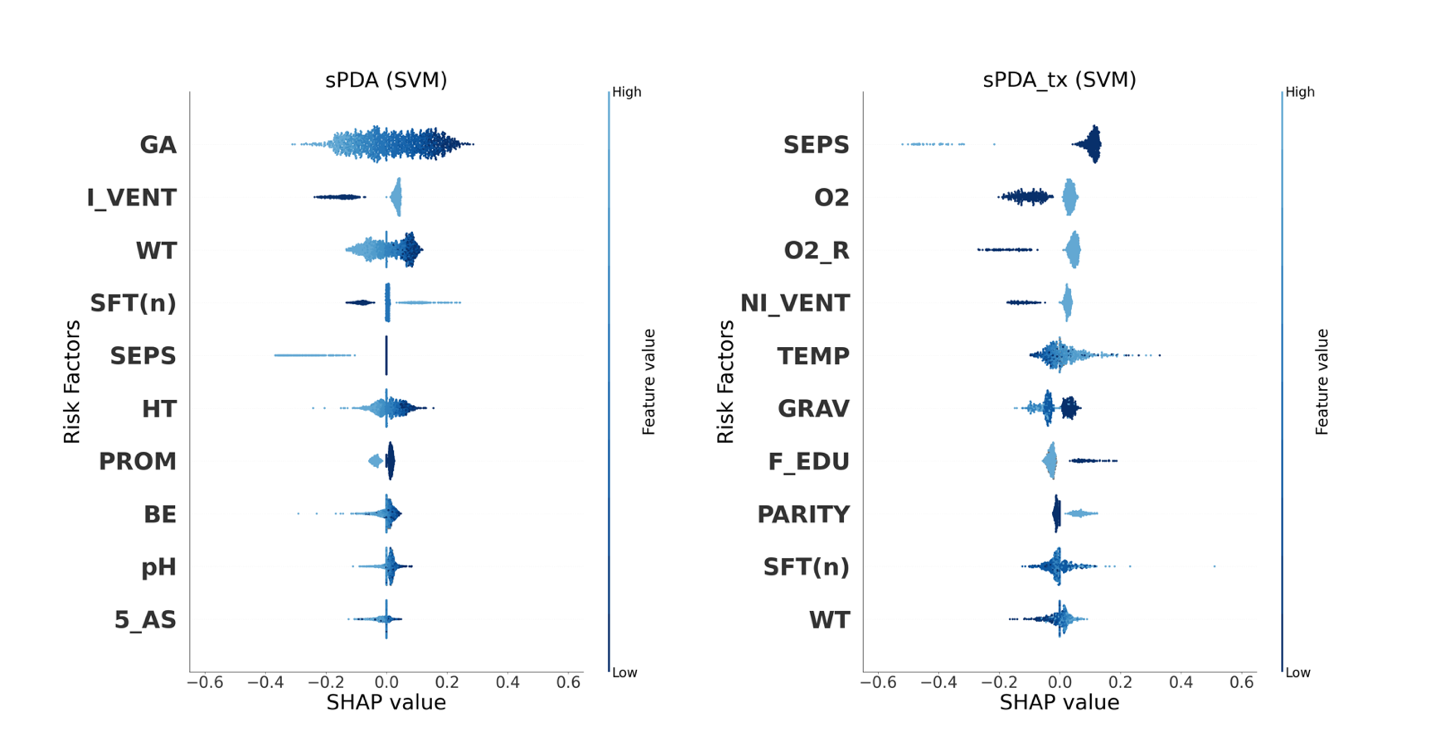


(e) k-NN


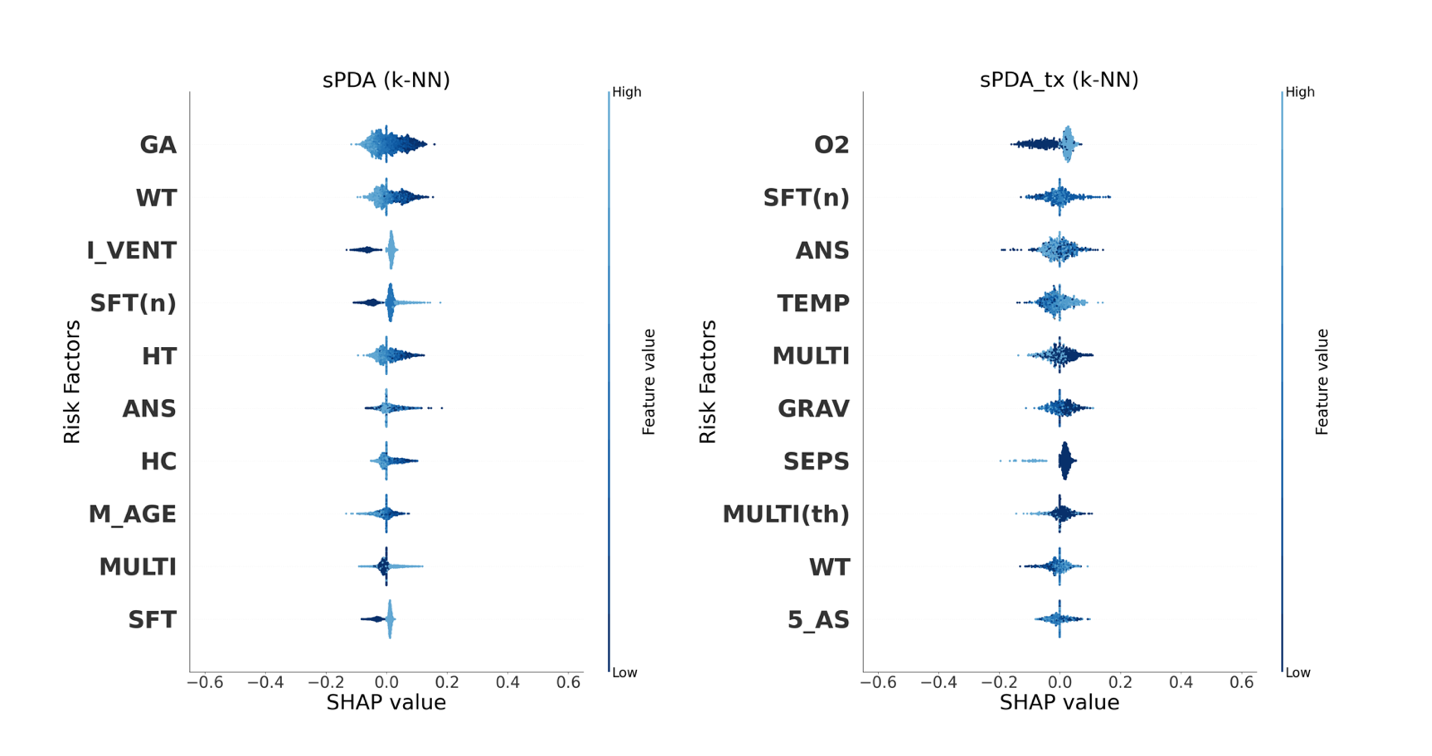


**Supplementary Figure 2**. Summary plots of the obtained SHAP values for sPDA and sPDA_tx prediction. SHAP contributions of the top 10 risk factors when (a) the RF, (b) the L-GBM, (c) the MLP, (d) the SVM and (e) k-NN are used to predict sPDA (left) and sPDA_tx (right). The X-axis represents the risk factor's contribution to the model's predictions (SHAP values). If a risk factor pushes a classifier to make a positive prediction, it is positioned to the right on the X-axis, and if it pushes the classifier toward a negative class, it is positioned to the left on the X-axis. The dots are plotted so that they do not overlap and are distributed vertically. The color bar on the right indicates the value of the corresponding risk factor, and the larger the value is, the brighter the color is. If the light-colored dots are positioned on the right side, we can interpret that the factors have positive relationships with sPDA or sPDA_tx in terms of model prediction. If the dark-colored dots are on the right, the relationship is interpreted as negative.

Abbreviations: PDA, patent ductus arteriosus; sPDA, symptomatic PDA; sPDA_tx, sPDA with any treatment; RF, random forest; L-GBM, light gradient boosting machine; MLP, multilayer perceptron; SVM, support vector machine; K-NN, k-nearest neighbors. The abbreviations of all factors are shown in Supplementary Table 1.
